# Gel-based and gel-free proteome data associated with controlled deterioration treatment of *Glycine max* seeds

**DOI:** 10.1016/j.dib.2017.09.056

**Published:** 2017-10-02

**Authors:** Cheol Woo Min, Seo Hyun Lee, Ye Eun Cheon, Won Young Han, Jong Min Ko, Hang Won Kang, Yong Chul Kim, Ganesh Kumar Agrawal, Randeep Rakwal, Ravi Gupta, Sun Tae Kim

**Affiliations:** a Department of Plant Bioscience, Life and Industry Convergence Research Institute, Pusan National University, Miryang 627-706, Republic of Korea; b National Institute of Crop Science, RDA, Miryang 627-803, Republic of Korea; c Research Laboratory for Biotechnology and Biochemistry (RLABB), GPO 13265, Kathmandu 44600, Nepal; d GRADE (Global Research Arch for Developing Education) Academy Private Limited, Adarsh Nagar-13, Birgunj 44300, Nepal; e Faculty of Health and Sport Sciences, University of Tsukuba, 1-1-1Tennodai, Tsukuba 305-8574, Ibaraki, Japan

## Abstract

Data presented here are associated with the article: “In-depth proteomic analysis of soybean (*Glycine max*) seeds during controlled deterioration treatment (CDT) reveals a shift in seed metabolism” (Min et al., 2017) [1]. Seed deterioration is one of the major problems, affecting the seed quality, viability, and vigor in a negative manner. Here, we display the gel-based and gel-free proteomic data, associated with the CDT in soybean seeds. The present data was obtained from 2-DE, shotgun proteomic analysis (label-free quantitative proteomic analysis) using Q-Exactive, and gene ontology analysis associated with CDT in soybean seeds (Min et al., 2017) [1].

**Specifications Table**

**Table:** 

Subject area	Biology
More specific subject area	Plant science, Proteomics, Controlled Deterioration Treatment (CDT)
Type of data	Tables and Figures
How data was acquired	MALDI-TOF/TOF-MS (ABI4800, Applied Biosystems, Framingham, MA, USA) and QExactive ^TM^ Orbitrap High-Resolution Mass Spectrometer (Thermo Fisher Scientific, USA) coupled with UHPLC Dionex UltiMate ® 3000 (Thermo Fisher Scientific, USA) system
Data format	Raw, Analyzed
Experimental factors	CDT in soybean seeds
Experimental features	CDT related proteins were characterized
Data source location	Department of Functional Crop, National Institute of Crop Science (NICS), Rural Development Administration (RDA), Miryang, South Korea (latitude 35N)
Data accessibility	Data within this article and the ProteomeXchange Consortium via the PRIDE [6] partner repository with the dataset identifier PXD006064

**Value of the data**

• The presented data show a shift in diverse metabolic processes in soybean seeds under CDT stress condition.
• A total of 1626 proteins were identified from label-free quantitative proteome analysis by Q-Exactive and 31 proteins from 2-DE under CDT.
• This data provide new evidences on CDT associated changes in low abundance proteins (LAPs) proteome profiles and metabolic process in soybean seeds.

## 1 Data

**Fig. 1:**
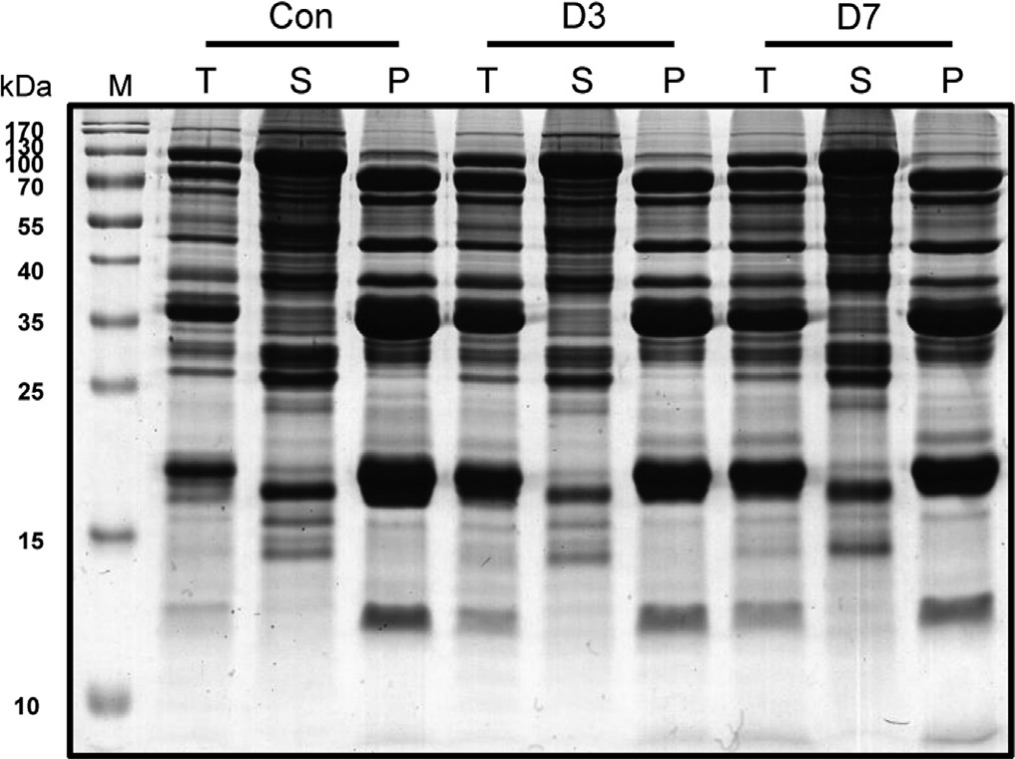
SDS-PAGE analysis of precipitated protein fraction using PSP method. Abbreviations: T-Total, S-PS supernatant, P-PS pellet.

**Fig. 2:**
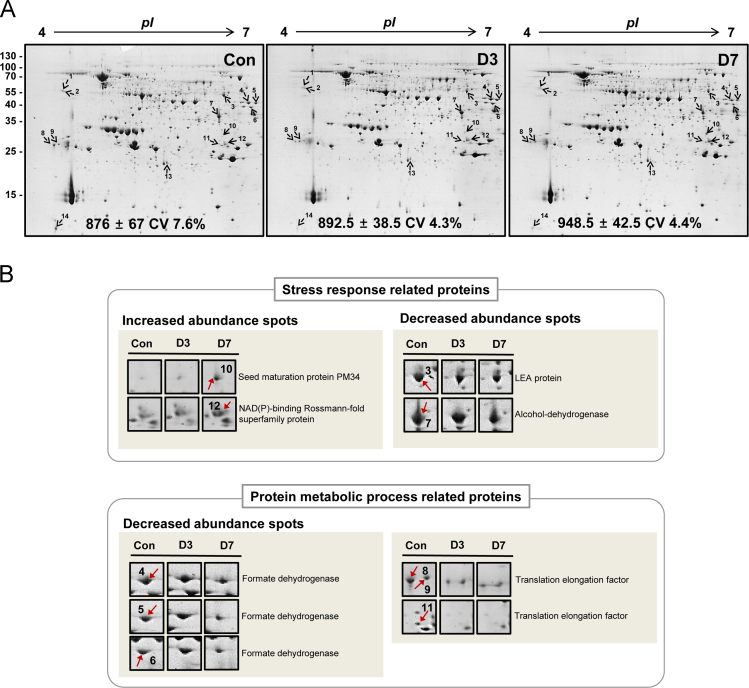
2-DE analysis of CDT seeds using 4–7 cm pH strip. (A) PSP method was applied and PS supernatant fraction was loaded for comparative analysis of the LAPs in CDT seeds. (B) Enlarged view of the major proteins related to stress response and protein metabolism.

The dataset reported here was obtained from the proteome analysis of CDT exposed soybean seeds, analyzed by gel-based (1-DE and 2-DE) and gel-free (label-free proteome) approaches (Fig. 1, Fig. 2). Supplementary Table 1–4 representatively show list of identified proteins from gel-based (Supplementary Table. 1) and gel-free (Supplementary Table 2–5) proteomic analysis. Furthermore, gene ontology and pathway analysis indicated major metabolic changes during CDT in soybean seeds [1].

## 2 Experimental design, materials and methods

### 2.1 Plant materials

Soybean seeds were collected from the experimental field of National Institute of Crop Science (NICS), Rural Department Administration (RDA) at Miryang, Korea. The soil was supplemented with a standard RDA N-P-K fertilizer (N-P-K=3-3-3.3 kg/10 acre). Seeds were harvested in October (average temperature 23.5±3.5 °C, average day length 12 h 17 min).

### 2.2 CDT treatment and protein extraction

CDT and protein extraction was carried out as described previously [2]. In brief, 25 g of soybean seeds were incubated at 99% relative humidity and 42 °C, placed and sealed inside of chamber after adding 200 mL water to maintain humidity and harvested 3 days and 7 days. Protein extraction was carried out using PSP method as described previously [3], [4]. Proteins from each fraction were first analyzed on 1-DE and for depletion of seed storage proteins and then used for further analysis (Fig. 1).

### 2.3 Two-dimensional electrophoresis and MALDI-TOF/TOF MS

The 2-DE with MALDI-TOF/TOF MS analysis were carried out as described previously [1], [5]. Briefly, protein samples were resuspended in the rehydration buffer containing 7 M urea, 2 M thiourea, 4% v/v CHAPS, 2 M DTT, and 0.5% v/v IPG buffer pH 4–7 (GE Healthcare, Waukesha, WI, USA). Protein concentration of PSS fraction was determined by 2D-Quant kit (GE Healthcare). Total 600 µg protein of each sample was loaded onto the 24 cm IPG strip (pH 4–7) as described previously (Min et al. 2017). Gels were stained by colloidal Coomassie Brilliant Blue (CBB) and distained with 30% (v/v) methanol twice. Furthermore, the protein spots on 2-D gels which were showed differentially modulated under CDT were detected using ImageMaster 2D Platinum software 6.0 (GE healthcare). Each protein spots showed differentially modulated were carried out statistical test using Tukey's post-hoc test (*p*-value <0.05, Fig. 2, and Supplementary Table 1). The selected spots were subjected in-gel digestion with trypsin and identified by MALDI-TOF/TOF MS (ABI4800, Applied Biosystems, Framingham, MA, USA) as described in details previously [1] (Supplementary Table 1).

### 2.4 Label-free quantitative proteome analysis with Q-Exactive

The isolated proteins were carried out label-free quantitative proteome analysis using QExactive^TM^ Orbitrap High-Resolution Mass Spectrometer (Thermo Fisher Scientific, USA) coupled with UHPLC Dionex UltiMate® 3000 (Thermo Fisher Scientific, USA) system as described previously [1]. In brief, CDT proteins were digested using in-solution tryptic digestion (filter-aided sample preparation, FASP) and further analyses were conducted as described in details previously [1] (Supplementary Table 2–4). The mass spectrometry proteomics data have been deposited to the ProteomeXchange Consortium via the PRIDE [6] partner repository with the dataset identifier PXD006064.

### 2.5 Statistical test and functional classification

Label-free quantitative proteome analysis was carried out by MaxQuant software (version 1.5.3.30) [1], [7] followed by statistical analysis of the obtained data using Perseus software (version 1.5.8.5) [8]. Multiple sample test was performed to find out significant differences (≥1.5 fold change, permutation based FDR <0.01) in the protein abundance during CDT (Supplementary Table 2–4).

## Appendix A Supplementary material

Supplementary material: List of CDT responsive proteins as identified by MALDI-TOF/TOF MS.

.

Supplementary material: Identification of differentially accumulated proteins by Q-Exactive Mass Spectrometer.

.

Supplementary material: List of degraded proteins during CDT.

.

Supplementary material: List of CDT induced proteins.

.

Supplementary material: List of total identified proteins along with their LFQ intensity values.
